# Antitumor efficacy of liposome-encapsulated NVP-BEZ 235 in combination with irreversible electroporation

**DOI:** 10.1080/10717544.2018.1444683

**Published:** 2018-02-27

**Authors:** Li Tian, Yang Qiao, Patrick Lee, Lucas Wang, Ashley Chang, Saisree Ravi, Thomas A. Rogers, Linfeng Lu, Burapol Singhana, Jun Zhao, Marites P. Melancon

**Affiliations:** a Department of Interventional Radiology, Division of Diagnostic Imaging, The University of Texas MD Anderson Cancer Center, Houston, TX, USA;; b College of Medicine, State University of New York Upstate Medical University, Syracuse, NY, USA;; c The University of Texas at Austin Dell Medical School, Austin, TX, USA;; d McGovern Medical School, Houston, TX, USA;; e Department of BioSciences, Rice University, Houston, TX, USA;; f Department of Chemistry, Mississippi State University, Starkville, MS, USA;; g Department of Chemical and Biomolecular Engineering, Rice University, Houston, TX, USA;; h Innovative Nanomedicine Research Unit, Chulabhorn International College of Medicine, Thammasat University, Rangsit Campus, Pathum Thani, Thailand;; i Department of Cancer Systems Imaging, The University of Texas MD Anderson Cancer Center, Houston, TX, USA;; j UT Health Graduate School of Biomedical Sciences, The University of Texas MD Anderson Cancer Center, Houston, TX, USA

**Keywords:** Nanoparticles, liposomes, irreversible electroporation (IRE), combination therapy, cancer, hepatocellular carcinoma, drug delivery, NVP BEZ-235

## Abstract

Irreversible electroporation (IRE) is an emerging minimally invasive tumor ablation technique that delivers short pulses of strong electric fields and kills cancer cells by disrupting their cell membranes with the electric pulses. However, clinical studies report that more than 10% of local tumor recurrences occur at the original ablated site. NVP BEZ-235 (BEZ) is a dual PI3K/mTOR inhibitor that has substantial anticancer effects. However, the clinical trials of BEZ was not satisfactory because of its low bioavailability and high toxicity, which stemmed from the use of oral administration of high doses over a long period of time. In this research, we prepared a liposomal formulation of BEZ (L-BEZ) for intratumoral injection and studied its antitumor efficacy alone and in combination with IRE. We hypothesized that IRE could release BEZ from the liposomes and that the combination could decrease tumor viability. Our results show that IRE released BEZ from its liposomal encapsulation. The combination of L-BEZ and IRE killed more Hep3B tumor cells *in vitro* than did L-BEZ or IRE alone and also inhibited cancer cell proliferation in nude mice bearing Hep3B xenografts. Combination of chemotherapeutic agent loaded nanoparticles could enhance the antitumor efficacy of IRE.

## Introduction

Exposure to electrical currents produces local defects in cell membranes, making them permeable to macromolecules and other chemical entities that otherwise require special transport mechanisms or are slow to passively diffuse across the cell membrane (Gehl, 2003). When the electric shocks have short durations, few pulses, and low electric fields, these local defects are transient. The cell membrane eventually reseals, a process known as reversible electroporation (Belehradek et al., 1993). Most electrochemotherapy for cancer uses parameters that cause reversible electroporation (Belehradek et al., 1993). However, local defects in the cell membrane may become permanent—and cells may die—if the electric shocks are of longer durations, have more pulses, and use higher electric fields, in a process called irreversible electroporation (IRE; Davalos et al., 2005). IRE is used as a nonthermal focal tumor ablation treatment (Davalos et al., 2005). Although IRE offers many advantages over surgery, such as the ability to ablate near major vessels, it also presents many challenges, including assessing tissue conductivity, determining the appropriate parameters, and positioning the electrode needles (Philips et al., 2013; Golberg et al., 2015; Miklavcic & Davalos, 2015). Moreover, the inherently heterogeneous structure of a tumor affects the impedance distribution, which in turn affects the electric field distribution and introduces uncertainty regarding the ablation outcome. Increasing the number of electrodes used may improve the precision of the computation of the electric field to be delivered. However, the positioning of multiple electrode needles lengthens the operation time and is challenging for clinicians (Miklavcic & Davalos, 2015).

Incomplete ablation and tumor recurrence are also reported after IRE (Philips et al., 2013). A recent clinical report on IRE treatment of soft-tissue tumors indicated that the tumor recurrence rate at 18 months was 31%, of which 10.7% were recurrences at the ablation site (Philips et al., 2013). Although no statistically significant relationships were found, the recurrences tended to occur in larger tumors, in areas with high vascular invasion, and in pancreatic lesions (Golberg et al., 2015). Another study examined the effects of IRE on rat livers, which have highly heterogeneous microstructure, chemical composition, and vasculature (Golberg et al., 2015). Cells close to large or clustered vessel structures were more apt to remain viable after IRE, an effect attributed to ‘electric field sinks,’ areas of reduced electric fields. Interestingly, these electric field sinks may offer an opportunity to incorporate the use of nanoparticles and electrochemotherapy.

Electrochemotherapy is a new technique that combines conventional chemotherapy with cell-membrane electroporation to enhance the transport of chemotherapy drugs into cells (Belehradek et al., 1993). Studies showed that intracellular drug concentrations increased by 300- to 700-fold in electroporated cells (Saczko et al., 2014) and that drug concentrations can remain elevated in the tumor area for several hours (Jarm et al., 2010). Nanoparticles are promising carriers for chemotherapy agents because of their many advantages over bare small molecules (Blanco et al., 2015). Nanoparticle-based treatments require less frequent dosing because they are generally more bioavailable than are standard agents (Gelperina et al., 2005). Chemotherapy agents delivered with nanoparticles have also been shown to have enhanced anticancer and fewer side effects than small molecular chemotherapy agents because they take advantage of the enhanced permeation and retention effect, which utilizes the leaky vessels and damaged lymphatic drainage in the tumor area and allows the accumulate of nanoparticles and the incorporation of targeting ligands, which allows specific targeting of the nanoparticles to tumor cells (Blanco et al., 2015). Finally, nanoparticles have potential theranostic applications, especially in combination with other clinical or interventional modalities (Tian et al., 2016).

In this study, we evaluated the anticancer effect of the combination of IRE and a liposomal formulation of NVP BEZ-235 (BEZ). BEZ is a synthetic imidazo[4,5-c]quinoline derivative compound that acts as a selective dual pan-class I phosphoinositide 3-kinase (PI3K) and mammalian target of rapamycin (mTOR) kinase inhibitor (Maira et al., 2008). It reversibly binds the ATP-binding sites of class I PI3K and mTOR kinases, inhibiting their catalytic activities. It has been shown to inhibit PI3K/Akt/mTOR signaling and to have antiproliferative and antitumor activity in several cancers, including breast cancer, glioma, lymphoma, non-small cell lung cancer, pancreatic cancer, and renal cell carcinoma (Cao et al., 2009; Liu et al., 2009; Bhende et al., 2010; Cho et al., 2010; Xu et al., 2011). Like other chemotherapeutic agents, BEZ needs to be delivered into the cytoplasm at a sufficient concentration to be effective. However, BEZ is highly hydrophobic and poorly soluble in water, so in clinical trials it was administered orally at a high dose over a long period of time. The efficacy of orally administered drugs is affected by many factors, including bioavailability (Chakraborty et al., 2009) and the first-pass effect (Zimm et al., 1983), and oral administration may not be feasible for cancer patients with dysphagia (Polk et al., 1967; Krech & Walsh, 1991). Clinical trials of BEZ showed toxicity that could be attributable to its having been delivered systemically. In a phase II trial involving 20 patients with locally advanced or metastatic transitional-cell carcinoma, 50% of the patients had grade 3–4 adverse events (Seront et al., 2016). Therefore, we developed a liposomal formulation of BEZ (L-BEZ) that could be injected systemically or intratumorally.

We hypothesized that L-BEZ improves the solubility of BEZ in aqueous solution and IRE releases BEZ from the liposome. Therefore, the combination of IRE and intratumoral injection of L-BEZ has better antitumor efficacy than IRE alone. To test this hypothesis, we evaluated the *in vitro* cytotoxicity of IRE, L-BEZ, and a combination of IRE and L-BEZ using Hep3B hepatocellular carcinoma cells. We also assessed tumor necrosis in Hep3B-xenograft mice treated with IRE, L-BEZ, or a combination. Our results confirmed that the combination treatment had the strongest antitumor effect and most effectively limited cancer-cell proliferation.

## Materials and methods

### Chemicals

1,2-Dipalmitoyl-sn-glycero-3-phosphocholine (DPPC), 1,2-distearoyl-sn-glycero-3-phosphoethanolamine-N-[amino(polyethylene glycol)-2000] (ammonium salt) (DSPE-PEG), and cholesterol were purchased from Avanti Polar Lipids Co. (Alabaster, AL) and used without further purification. NVP-BEZ235 (BEZ) was purchased from LC Laboratories (Woburn, MA,). All other chemicals were purchased from Sigma-Aldrich (St. Louis, MO ) and were used without further purification.

### Liposome preparation

Liposomes were prepared using the hydration-sonication method with an optional extrusion step (Samad et al., 2007). Briefly, DPPC, DSPE-PEG, cholesterol, and BEZ were dissolved in chloroform: methanol 4:1 (v/v) and were vacuum-dried on a rotary evaporator at 49 °C to form a thin film. The thin film was then hydrated in HEPES-buffered saline (HBS) solution. To obtain the liposome solution, 20 rounds of hydration-sonication cycles at 60 °C were performed. Free BEZ was removed by passing the liposome solution through a Sephadex G-25 column (GE Healthcare, Buckinghamshire, UK) to obtain the final BEZ liposome solution (L-BEZ). Blank liposomes were prepared similarly but without BEZ. Particle size was monitored by measuring dynamic light scattering at a scatter angle of 90° using a Brookhaven particle-size analyzer (Holtsville, NY). All liposome solutions were stored at 4 °C and used within a week of production.

### BEZ quantification

The amount of BEZ in L-BEZ was quantified on a ClarioStar plate reader (BMG Labtech, Ortenberg, Germany) with the excitation wavelength at 325 nm and emission wavelength at 425 nm. BEZ was released from L-BEZ by adding two parts dimethyl sulfoxide (DMSO) to one part L-BEZ by volume. The mixture was vortexed and cooled to room temperature. The blank liposome was processed the same way and was used as a blank control. BEZ standards were prepared by dissolving BEZ in DMSO:HBS (2:1, v/v) at different concentrations.

### Cell culture and L-BEZ cytotoxicity assays

Hep3B human hepatocellular carcinoma cells (purchased from ATCC) were cultivated in Dulbecco’s modified Eagle’s medium (DMEM) supplemented with 10% fetal bovine serum (F0926-500, Sigma) and 1% penicillin-streptomycin (MT-30-002-CI, Mediatech). Cell cultures were maintained at 37 °C and 5% CO_2_. For cytotoxicity studies, the cells were plated in 96-well plates. Twenty-four hours after the cells were plated, BEZ and L-BEZ were diluted in DMEM to various final concentrations and were added to the cells. After another 72 hours of incubation, cytotoxicity was examined using a 3-(4,5-dimethylthiazolyl-2)-2,5-diphenyltetrazolium bromide (MTT) assay (M6494, Thermo Fisher Scientific Co.,Waltham, MA). Corresponding blank liposomes were also tested. Cell viability curve was plotted in Microsoft Excel (Microsoft, Redmond, WA) and the IC50 values were read from the curve.

### In vitro *electroporation and cell viability assays*

*In vitro* electroporation was carried out using a BTX ECM 830 electroporation system (Harvard Apparatus, Holliston, MA). Hep3B cells were subjected to electroporation at field strengths ranging from 0 to 2500 V/cm. The electroporated cells were then plated in 96-well plates. An MTT assay was conducted 72 h after electroporation to assess cell viability.

For the study of BEZ release from L-BEZ by IRE, the liposomes were electroplated at 2500 V/cm. The supernatant was collected after centrifuging at 10,000 rpm for 5 min. Then the supernatant was mixed with the cell culture medium at 1:9 (v:v), so there was 10% supernatant in the cell culture medium. The cell culture medium containing the supernatant was added to the cells. Blank liposomes were electroporated and the supernatant was collected, mixed with the cell culture medium, and were added to the cells following the same procedure. MTT assay was carried out 72 h later.

For combination therapy with BEZ or L-BEZ, IRE was performed 3 min before, at the same time as, or 1.5 min after the addition of BEZ or L-BEZ. Then MTT assay was carried out 72 h later.

### Effects of IRE and L-BEZ on xenograft tumors in mice

We determined the acute effects of treatment with IRE in combination with L-BEZ using female nude mice bearing Hep3B xenografts. Female nude mice (nu/nu, 4–5 weeks old) were purchased from the Experimental Radiation Oncology Breeding Core in MD Anderson Cancer Center. The protocol was approved from the Institutional Animal Care and Use Committee (Protocol Number 00001234-RN01). Three million Hep3B cells in PBS: Matrigel (Corning, Corning, NY) 1:1 were inoculated on the right hind leg of each mouse. Treatment started when the tumor size reached 8 mm to mimic incomplete electroporation. The mice were randomly allocated into four treatment groups with four mice per group: control (no treatment), IRE alone, L-BEZ alone, and IRE + L-BEZ. Mice in the IRE group received one dose of IRE at 2500 V/cm, with 99 pulses at 1 s intervals. In the L-BEZ group, the mice received a single intratumoral injection of 100 μL L-BEZ (equivalent to 0.1 mg BEZ). In the IRE + L-BEZ group, the mice received a single dose of L-BEZ and then a single dose of IRE 90 s later. The animals were anesthetized with isoflurane during treatment and returned to their cages when they had fully recovered from anesthesia and treatment. Seventy-two hours after treatment, all animals in four groups were euthanized in a carbon dioxide (CO_2_) chamber followed by cervical dislocation and tumors were extracted. Hematoxylin and eosin (H&E) staining was performed by the Department of Veterinary Medicine & Surgery in MD Anderson Cancer Center. The stained slides were scanned using Aperio ScanScope® CS slide scanner (Aperio Technologies, Vista, CA) and Aperio ImageScope (v12.1.0.5029, Aperio Technologies, Vista, CA; http://www.aperio.com/download.asp) software was used to delineate the necrotic area. Percentage necrosis was calculated by dividing the necrotic area by the total area of the tumor multiplied by 100. Adjacent slides were stained using TACS® 2 TdT DAB *in Situ* Apoptosis Detection Kit (4810-30-K) from Trevigen^®^ for terminal deoxynucleotidyl transferase (TdT) dUTP Nick-End Labeling staining, or TUNEL assay to determine apoptosis. Percentage apoptosis was calculated by dividing the apoptotic area by the total area of the tumor multiplied by 100. Ki-67 staining was employed for cell proliferation analysis using Ki-67 (D2H10) rabbit monoclonal antibody (catalogue number 9027S, Cell Signaling Technology, Inc., Danvers, MA 01923), Dako LSAB2 System-HRP (catalogue number K0675), and Dako Liquid DAB + Substrate Chromogen System (catalogue number K3468) (both purchased from Agilent Technologies, Inc., Santa Clara, CA 95051). The Ki-67 positive (brown) nuclei and the Ki-67 negative (blue) nuclei were counted. Total nuclei was calculated as the sum of Ki-67 positive and negative nuclei. Percentage of Ki-67 positive cells was calculated by dividing the number of Ki-67 positive nuclei by the number of total nuclei multiplied by 100.

For the long-term antitumor efficacy study, the mice underwent xenograft implantation and were grouped and treated as described above. The tumor size was measured by calipers for two weeks every 2–3 days after treatment. The mice were then sacrificed and the tumors were harvested and stained for H&E. The slides were scanned and analyzed using Aperio eSlide Manager as described previously.

### Statistical analysis

One-way analysis of variance (ANOVA) followed by *post-hoc* test (individual *post-hoc* test is specified in the ‘Results’ section) was used to compare the results. SigmaPlot software (Systat, San Jose, CA) and Microsoft Excel (Microsoft, Redmond, WA) were used for statistical analysis. *p* values less than .05 were considered significantly different for *in vitro* experiments, and *p* values less than .1 were considered significantly different for *in vivo* experiments (Dahiru, 2008).

## Results

### Preparation and characterization of L-BEZ

**Figure 1.**
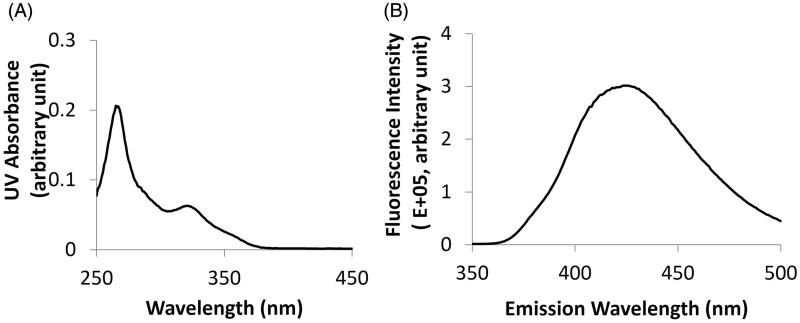
Quantification of BEZ. (A) The UV absorbance spectrum showed an absorbance peak at 325 nm. (B) The fluorescence spectrum showed an emission peak at 425 nm when BEZ solution was excited at 325 nm.

The particle size of L-BEZ was 510.4 ± 15.8 nm with a polydispersity index (PDI) of 0.005. BEZ showed an absorbance peak at 325 nm on the ultraviolet spectrum (Figure 1(A)) and an emission peak at 425 nm when the excitation wavelength was set at 325 nm (Figure 1(B)), so BEZ quantification was performed using a fluorescence plate reader at an excitation wavelength of 325 nm and emission wavelength of 425 nm. The loading efficiency was 95% when 0.4 mg of BEZ was added to the formulation. The BEZ concentration in the synthesized liposome was 0.11 mg/mL, with a final volume of 3.5 mL. By adjusting the BEZ feeding amount, the BEZ concentration could be made to reach as high as 2.7 mg/mL.

Since liposomes have a similar structure to cell membranes, we determined whether electroporation could release the BEZ inside the liposome. We subjected solution of L-BEZ to electroporation using field strengths ranging from 0 to 2500 V/cm. The L-BEZ particle size was 102.9 ± 2.6 nm before electroporation, with a narrow PDI of 0.085. When the liposomes were disrupted after electroporation, PDI will widen and multiple peaks on the particle size measurement was observed (Supplementary Figure S1). This is due to the disruption of the particle membrane after electroporation. Phase separation was also observed.

In addition to the ability of electroporation to disrupt the membrane-like structure of the liposome, we also examined the cytotoxicity of the drug released from L-BEZ by electroporation. Blank liposomes and L-BEZ were electroporated at 2500 V/cm and the supernatants were collected and added to the Hep3B cells (Supplementary Figure S2). MTT assays showed that the percentage of viable cells decreased from 80.12% in cells treated with the blank liposome to 48.97% in cells treated with L-BEZ (*p* < .05, one-way ANOVA).

### In vitro *cytotoxicity assays of L-BEZ, IRE, and the combination of IRE and L-BEZ in Hep3B cells*

**Figure 2.**
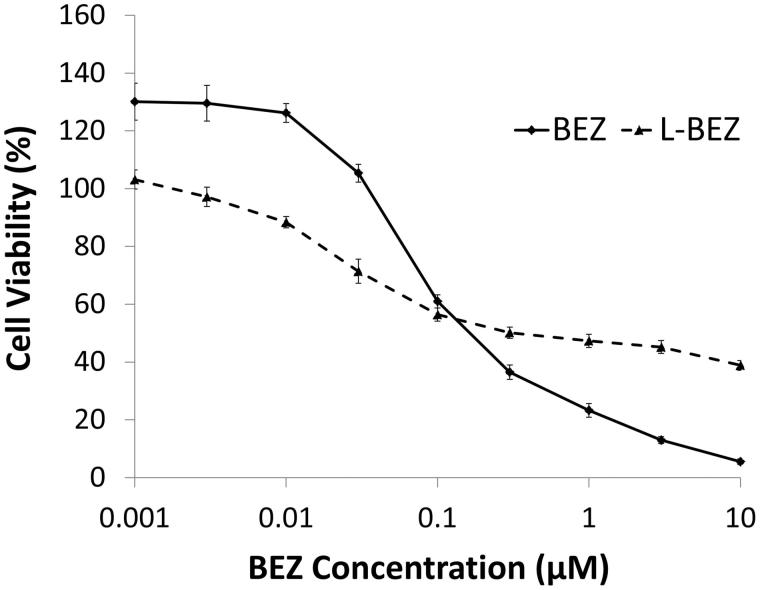
Cytotoxicity of BEZ and L-BEZ. The IC_50_ values of BEZ and L-BEZ were 0.1 and 0.3 µM, respectively, on Hep3B cells by MTT assay. Untreated cells were also tested and the viability was 100% for both groups.

The cytotoxicity curves of BEZ and L-BEZ are shown in Figure 2. BEZ alone had a half maximal inhibitory concentration (IC_50_) value of 0.1 µM. L-BEZ had a slightly higher IC_50_ valueof 0.3 µM, and was slightly less cytotoxic at higher concentrations than BEZ alone.

**Figure 3.**
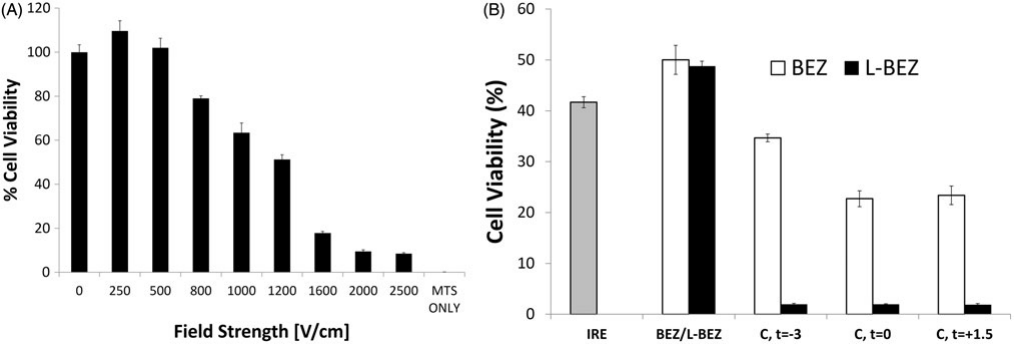
Effect of Electroporation and L-BEZ on Cells. (A) Cell viability after electroporation at different field strengths. More cells were viable after electroporation at 250 V/cm than with no electroporation. (B) Cell viability after treatment with IRE in combination with L-BEZ at various intervals. Combination therapy (abbreviated as C) is BEZ or L-BEZ following IRE treatment at 1600 V/cm, 100 ms, and 99 pulses to the cell. The time interval between IRE and BEZ or L-BEZ treatment is indicated (t = −3, 0, and +1.5 min, respectively).

We found that increasing the electroporation field strength decreased the viability of Hep3B cells (Figure 3(A)). However, more viable cells were found in samples electroporated at the lowest tested field strength (250 V/cm) after 72 h than were found in non-electroporated samples. Approximately 10% of the electroporated cells remained viable even at the highest field strength (2500 V/cm).

We also evaluated the cytotoxic effects of the combination of IRE and L-BEZ on Hep3B cells (Figure 3(B)). The combination of IRE and BEZ or L-BEZ was more cytotoxic than was IRE alone. A comparison of IRE + BEZ and IRE + L-BEZ showed that L-BEZ was more cytotoxic than BEZ regardless of whether L-BEZ or BEZ was added 3 min before, at the same time, or 1.5 min after electroporation. This evidence supported our hypothesis that the combination of IRE and L-BEZ is more cytotoxic than the combination of IRE and BEZ.

### In vivo *effects of the combination of IRE and L-BEZ on tumor necrosis, apoptosis, and cell proliferation*

**Figure 4.**
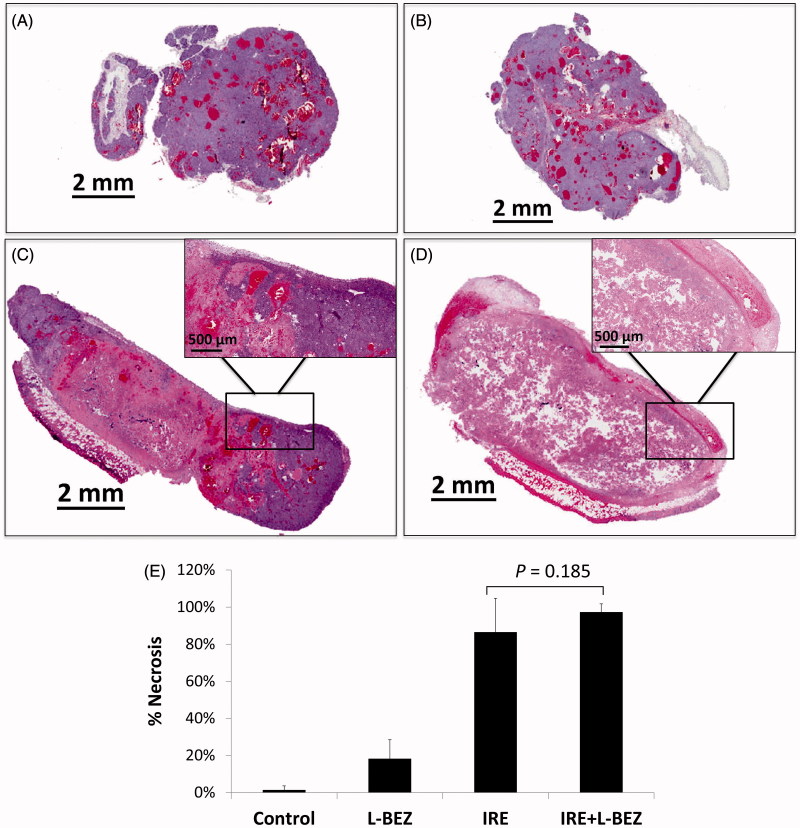
Tumor necrosis three days after treatment. Representative H&E stained tumor sections from the (A) control group; (B) L-BEZ group; (C) IRE group; and (D) IRE + L-BEZ group. The tumors treated with IRE and IRE + L-BEZ had higher mean necrosis percentages than did the tumors in the other groups. The insets in (C) and (D) illustrate that live tumor cells were found within the margin of the tumors, while the necrotic cells were found in the center of the electroporated area. No living cells were observed in the IRE + L-BEZ-treated tumors. Tumor size at the start of the treatment: IRE, 11.6 × 10.6 mmand IRE + L-BEZ, 11.3 × 11.4 mm. (E) Comparison of percentage of necrosis by H&E staining (one-way ANOVA followed by Holm-Šidák *post-hoc* analysis, *p* values between: CTL and LBEZ, .094; CTL and IRE, <.001; CTL and IRE + LBEZ, <.001; LBEZ and IRE, <.001; LBEZ and IRE + LBEZ, <.001; and IRE and IRE + LBEZ, .185).

To assess the antitumor effects of IRE and L-BEZ *in vivo*, we assessed necrosis three days after treatment in tumor from xenograft-bearing mice in the four treatment groups described above (Figure 4). Representative H&E-stained slides from tumors treated with IRE or IRE + L-BEZ are shown in Figures 4(C,D). These two tumors were chosen because they had a similar size at the time of initial treatment (11.6 × 10.6 mm and 11.4 × 11.3 mm for IRE and IRE + L-BEZ group, respectively). The inset of Figure 4(C) illustrates necrosis in the center of the tumor, around the center of the electroporated area, where the strongest electroporation was delivered. Live cells were detected in the IRE-treated tumor, particularly at the margins of the tumor. In comparison, no living cells were observed within the margin of the tumor treated with IRE + L-BEZ (Figure 4(D)). These data suggest that in tumors of the same size, treatment with the combination of L-BEZ and IRE increases tumor necrosis over treatment with IRE alone.

The mean percentage of necrosis in each treatment group is as follows: control group 1.32 ± 2.29, L-BEZ group 18.2 ± 10.3, IRE group 86.4 ± 18.4, and IRE + L-BEZ group 97.2 ± 4.59%, respectively. The results were statistically analyzed using one-way ANOVA followed by the Holm-Šidák correction for multiple comparisons. The IRE and IRE + L-BEZ groups both had higher mean necrosis percentages than did the L-BEZ group (*p* < .001). The IRE + L-BEZ group had the highest mean percentage necrosis; however, the difference between the mean percentage necrosis in IRE + L-BEZ group and the IRE group was not statistically significant (*p* = .185; (Figure 4(E)).

**Figure 5.**
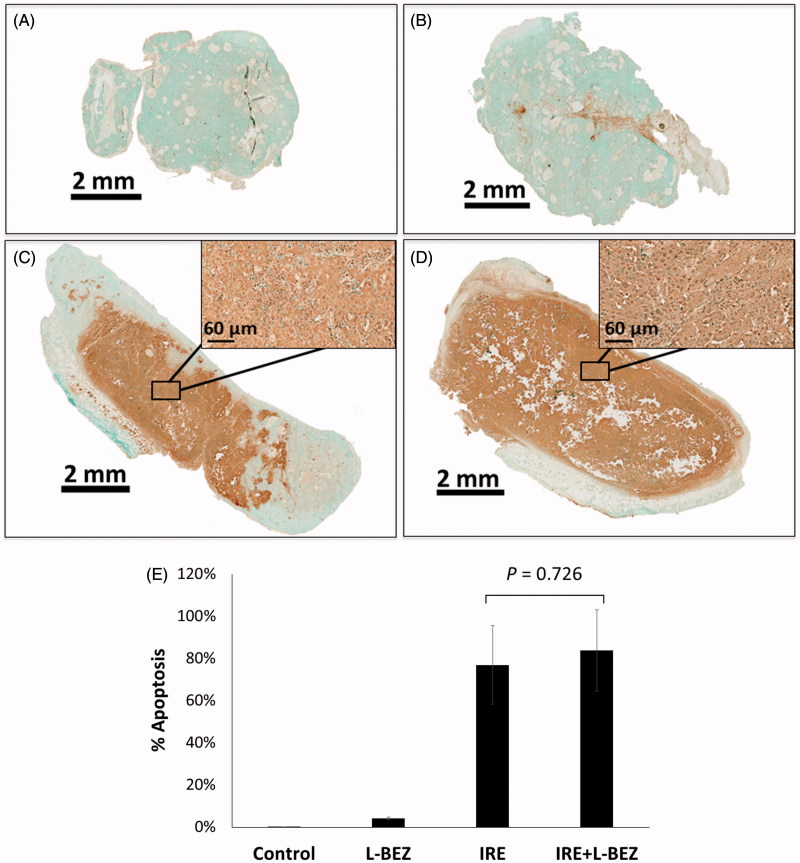
Tumor apoptosis three days after treatment using TUNEL Staining. Representative tumor sections from the (A) control group; (B) L-BEZ group; (C) IRE group; and (D) IRE + L-BEZ group. The tumors treated with IRE and IRE + L-BEZ had higher mean apoptosis percentages than did the tumors in the other groups. The insets in (C) and (D) illustrate that the dark apoptosis-positive signal was not confined in the nuclei but spread out to the cytosol and in some intratumoral space. (E) Comparison of percentage of apoptosis by TUNEL staining (one-way ANOVA followed by Holm-Šidák *post-hoc* analysis, *p* values between: CTL and LBEZ, .671; CTL and IRE, < .001; CTL and IRE + LBEZ, <.001; LBEZ and IRE, <.001; LBEZ and IRE + LBEZ, <6.001;and IRE and IRE + LBEZ, .726).

Apoptosis was evaluated by TUNEL staining (Figure 5). For the IRE and IRE + L-BEZ groups, the staining showed a diffused pattern, i.e. the dark brown signal was not confined in the nuclei but spread out to the cytosol and in some intratumoral space (Figure 5(C,D) and their insets). The mean percentage of apoptosis in each treatment group is as follows: control group 0.03 ± 0.1, L-BEZ group 4.16 ± 0.6, IRE group 76.9 ± 18.6, and IRE + L-BEZ group 83.9 ± 19.3%, respectively. The IRE + L-BEZ group had the highest mean percentage apoptosis. However, the difference between the mean percentage of apoptosis in the IRE + L-BEZ group and the IRE group was not statistically significant (*p* = .726; Figure 5(E)).

**Figure 6.**
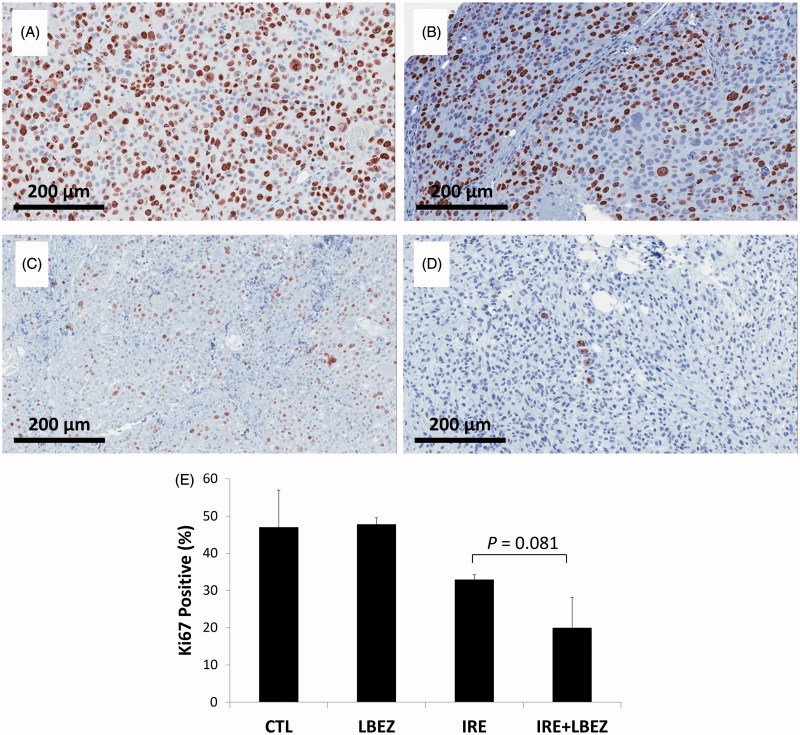
Ki-67 staining. Representative Ki-67- stained images of the center of the (A) control group; (B) L-BEZ group, showing nonproliferating cells near the injection route; C) IRE group, showing a marked decrease in the number of proliferating cells; and (D) IRE + LBEZ group, showing a marked decrease in the number of proliferating cells. (E) Comparison of the percentage of Ki-67–positive proliferating cells in the different treatment groups (one-way ANOVA followed by Holm-Šidák *post-hoc* analysis, *p* values between: CTL and LBEZ, .886; CTL and IRE, .086; CTL and IRE + LBEZ, .005; LBEZ and IRE, .091; LBEZ and IRE + LBEZ, .005; and IRE and IRE + LBEZ, .081).

Ki-67 staining was then used to detect proliferating cells (Figure 6). In the L-BEZ group, some nonproliferating cells were found along the injection site, where the L-BEZ had been introduced. The mean percentage of Ki-67-positive proliferating cells did not significantly differ between the L-BEZ and control groups (47.8 ± 1.7 and 47.0 ± 9.9%, respectively, *p* = .886). IRE treatment decreased the mean percentage of Ki-67-positive cells to 32.9 ± 1.3%, which was significantly lower than that found in the control (*p* = .086) and L-BEZ groups (*p* = .091). At 3 days after treatment, proliferating cells were found in the electroporated areas which corresponded to the necrotic areas on H&E-stained slides (i.e. the center of IRE treatment). Most proliferating cells were detected at the margins of the electroporated tumors, whereas most live cells were found in the necrosis areas (Figure 4). Interestingly, the mean percentage of proliferating cells in the margins of the IRE treated tumors was 54.5 ± 3.6%, which is higher than that of the control (no treatment, 47.0 ± 9.9%). IRE + L-BEZ treatment further lowered the percentage of proliferating cells to a mean of 20.0 ± 8.2%, which was significantly lower than the rate found in the IRE group (*p* = .081< .1). Higher rates of cell proliferation were found in the margins of some tumors, but the mean proliferation rate in the margins of IRE + L-BEZ was 47.7 ± 2.2% and it dropped to the nontreatment level in the control group (47.0 ± 9.9%), compared to the mean percentage of proliferating cells in the margins of the IRE treated tumors (54.5 ± 3.6%).

### *Long-term* in vivo *effects of the combination of IRE and L-BEZ on tumor growth and necrosis*

**Figure 7.**
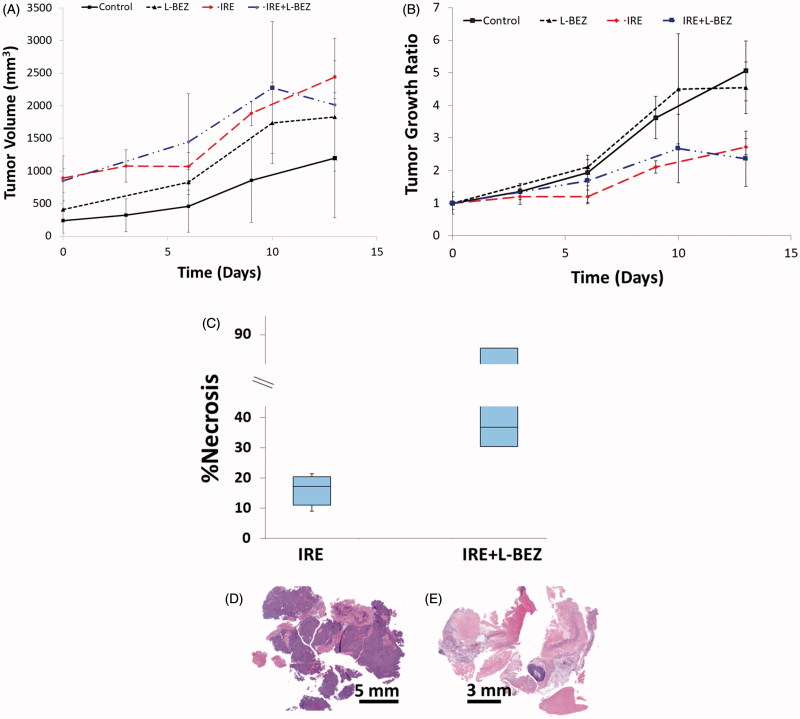
*In Vivo* antitumor effect of IRE in combination with chemotherapy on nude mice bearing Hep3B xenografts After one treatment. (A) Tumor size over time. (B) Tumor growth ratio over time. Both the IRE and IRE + L-BEZ groups had significantly lower tumor burden than did the control group (one-way ANOVA, *p* < .05). No statistically significant difference was observed between the IRE and IRE + L-BEZ groups (*n* = 4 mice per group) (C) Percentage of necrosis as quantified by H&E staining two weeks after one treatment. The median percentage necrosis of combination treatment with IRE + L-BEZ doubled that of IRE treatment alone. Representative H&E-stained slides of tumor treated with IRE alone (D), or IRE + L-BEZ (E). Notice the extent of necrosis exhibited in the tumor treated with the combination therapy compared to that in the tumor treated with IRE alone.

We monitored tumor size in mice with xenografts for two weeks after single treatment with IRE, L-BEZ, or IRE + L-BEZ. The tumor volume over time is reported in Figure 7(A). Due to the variation in the initial tumor size on the day of treatment, the relative tumor growth ratio was calculated and plotted in Figure 7(B). Both IRE and IRE + L-BEZ treatments stabilized tumor size for about one week after treatment (Figure 7(B)). Faster tumor growth was observed in all 4 groups after one week regardless of treatment. At the end of week 2, both the IRE and IRE + L-BEZ groups had significantly smaller tumors than did the control and L-BEZ groups (one-way ANOVA, *p* < .05). However, no significant difference in tumor burden was observed between the control and L-BEZ groups or between the IRE and IRE + L-BEZ groups.

Two weeks after treatment, the tumor masses were extracted, and H&E staining was performed to evaluate tumor necrosis (Figure 7(C–E)). The median percentage of necrosis in tumors treated with the combination of IRE + L-BEZ was twice that of tumors treated with IRE alone. However, no statistically significant difference was observed between the IRE and IRE + L-BEZ groups in terms of tumor size or percentage of necrosis at the end of the second week after treatment (*p* = .683).

## Discussion and conclusion

Liposomes are a well-studied nanoparticle formulation that can carry both hydrophobic and hydrophilic therapeutic molecules (Allen & Cullis, 2013). The lipid composition of a liposome affects its properties. For our experiments, the DSPE-PEG molar ratio was kept at 5 mol % because research has shown that 5–10 mol % of PEG-phospholipid can stabilize spherical liposomes (Edwards et al., 1997). Formation of an aggregate structure was observed when the PEG-phospholipid ratio exceeded 10 mol % and mixed micelles, not liposomes, were formed when the PEG-phospholipid ratio exceeded 15 mol % (Edwards et al., 1997). In one attempt using 20 mol % of DSPE-PEG, the extrusion resistance was extremely high. Thus, we kept the DSPE-PEG ratio at 5 mol %.

During the liposome optimization, we also prepared smaller L-BEZ particles of around 100 nm by extrusion. However, after extrusion, the BEZ loading efficiency dramatically dropped to 11 to 14% from over 90% before extrusion. Indeed, the loading efficiency of hydrophobic drugs in liposomes is known to be low (Campbell et al., 2001). Hydrophobic drugs are loaded within the lipid bilayer. Owing to the comparatively rigid structure and limited space of the bilayer, especially its decreased size and increased curvature after extrusion, the amount of hydrophobic drug molecules that can be incorporated into the bilayer is limited.

One commonly used strategy to increase the loading of hydrophobic drugs is the inclusion of small amounts of amphiphilic molecules in the formulation (Yang et al., 2007). However, since the present study is aimed to provide a proof-of-concept on the feasibility of combining nanoparticle-carrying chemotherapeutics with IRE, we fabricated a liposome with the highest possible BEZ loading. Thus, L-BEZ of around 500 nm was chosen for all of the experiments, if not otherwise noted.

We found that L-BEZ was less cytotoxic at higher concentrations. This result was not surprising, because BEZ has a higher affinity to lipids than to the aqueous cell culture medium. The structure of larger liposomes is usually multilamellar (Pabst et al., 2000), which means that more BEZ molecules are trapped in the bilayers inside larger particles and have poorer diffusion to the bulk aqueous solution. Thus, at higher concentrations, the effective amount of BEZ that enters the cell would decrease, resulting in weaker cytotoxic effects.

The liposome membrane and cell membrane are both lipid bilayers. Because of this structural similarity, we anticipated that electroporation would induce a certain degree of liposome destruction. Our results demonstrated that the liposome membrane was indeed disrupted by electroporation, even at the lowest applied field strength (250 V/cm) (Supplementary Figure S1), and that BEZ was released during the destruction of the liposome (Supplementary Figure S2). These results confirm our hypothesis that the release of BEZ from the liposomes triggered by electroporation increased the antitumor efficacy of BEZ.

When we assessed the effect of electroporation on cells, we noted that more cells remained viable after electroporation at 250 V/cm than after the control (no electroporation). This finding agreed with that of our *in vivo* studies showing that viable cells were mostly found at the periphery of the tumor - farthest from the center of the electroporation probe and where the lowest electric field was received. However, the combination of L-BEZ with electroporation significantly increased cytotoxicity. Literature suggests that the timing of electroporation and cell exposure to nanoparticles resulted in different levels of the cellular uptake of iron nanoparticles (West et al., 2013). Our *in vitro* result of IRE + L-BEZ suggests similar cytotoxicity regardless of timing. This could be due to the disruption of L-BEZ and the release of BEZ from L-BEZ by IRE. Thus, the cytotoxicity results from the combination of liposome-carrying BEZ plus the released drug, and not only from the nanoparticle (L-BEZ).

Our *in vivo* studies demonstrated that necrosis radiated out from the center of the tumor, surrounding the needle probe (Zhao et al., 2015), where the highest electroporation field strength was received. Live cells were detected within the margins of IRE-treated tumors, confirming our expectation that as it moves away from the center of IRE treatment, the electric field drops, and cells no longer die from the electric shock. In comparison, no living cells were observed in the IRE + L-BEZ-treated tumor as shown in Figure 4(D). These data suggest that combining L-BEZ with IRE decreases cancer cell viability and the potential for recurrence post-IRE ablation.

TUNEL staining detects double-strand DNA breakage. Al-Sakere et al. (2007) monitored tumor apoptosis after irreversible electroporation using TUNEL assay and found that TUNEL positive staining was localized within the nuclei at 5 min after the electroporation. However, diffusion of TUNEL staining from the nuclei was observed at 1 h after electroporation and by the end of 24 h, the TUNEL staining completely diffused from the nuclei to the entire tumor section. This phenomenon was characteristic of irreversible electroporation as this treatment breaks down the cell’s natural membrane barrier. TUNEL staining patterns of our tissue samples concur with Al-Sakere et al.’s results. However, our mice were not sacrificed until 72 h after IRE treatment and a more diffused TUNEL staining was observed. Thus, although TUNEL staining confirmed the involvement of apoptosis in the cell death following both IRE and IRE + lL-BEZ treatments, the quantification may not be able to provide a precise evaluation of the extent of apoptosis.

Golberg et al.( 2015) electroporated normal liver tissues and discovered incomplete electroporation near major vessels and cells recovered as soon as three days after electroporation. Thus, we performed Ki-67 staining to identify surviving tumor cells three days after treatment. The hotspots in Ki-67 staining overlapped with the hotspots in H&E staining and the strongest signals were generally found in the periphery of the tumors. However, we found that even in the necrotic areas of treated tumors, the cells began to recover viability three days after electroporation. The tumors in control and L-BEZ groups had similar viability. L-BEZ treatment did not result in low viability, which could be due to the slow release of BEZ from the liposome caused by BEZ’s high hydrophobicity. The percentage of viable cells in the IRE group significantly decreased compared to untreated controls, demonstrating the efficacy of IRE treatment. The combination of IRE and L-BEZ treatment produced the lowest percentage of viable cells of all the treatments, suggesting that the ablation and release of BEZ could enhance the anti-tumor efficacy of IRE. Thus, treatment with the combination of IRE and L-BEZ may kill tumors more efficiently than either single treatments and may decrease the risk of recurrence at the ablation site.

We also observed that in IRE group, the hot spots at the margins of the tumor had a slightly higher rate of cell proliferation, which agreed with the finding that cells electroporated at 250 V/cm had higher viability rates than untreated cells.

When we examined whether the length of treatment could have an effect on tumor growth, we found that tumor size was stable in both the IRE and IRE + LBEZ groups for about a week after treatment, after which we observed measurable tumor growth in both groups. After two weeks, the mean percentage necrosis in tumors treated with IRE + LBEZ was higher than that in tumors treated with IRE, but the difference was not statistically significant. However, we were not able to keep the mice longer than two weeks because of the increased growth of the Hep3B xenograft. In order to simulate a situation of late stage hepatocellular carcinoma, we started IRE and IRE + L-BEZ treatments when the Hep3B xenografts were at least 8 mm in one dimension. However, in our experience, these Hep3B xenografts grow aggressively when the size approached or exceeded 10 mm and this aggressive growth limits how long we could observe the treatment effects. Thus, these results suggest that more studies are needed to determine how to optimize dosing regimens to maximize the efficacy of the combination treatment at preventing recurrence at the ablation site. A different cancer cell line may also be needed to study the long-term effect of this combination treatment.

To the best of our knowledge, this is the first study that proved a higher *in vivo* efficacy of IRE in combination of a liposomal formulation of a chemotherapy agent when compared to IRE alone. However, the reason as to why electroporation at lower field strength could increase cell viability still needs investigation. Similar result was also observed by Silve et al. (2016) but is not discussed in their paper. It could potentially be from the extracellular/intracellular material change after electroporation, disruption of subcellular membranes and the downstream biological effect, activation of signaling pathways, or a combination of more than one factor. Understanding of this mechanism would be helpful in the development of precise targeting treatment to prevent tumor recurrence.

In summary, BEZ was encapsulated within the liposome, and electroporation effectively released BEZ from the liposome. The combination of IRE and L-BEZ significantly decreased cancer cell viability *in vitro* and proliferation *in vivo* within the electroporated area. The combination of IRE and L-BEZ could decrease the potential for tumor recurrence at the ablation site.

## Geolocation information

1515 Holcombe Blvd, Houston, TX, USA 77030

Latitude: 29.707198; Longitude: -95.396894

## Supplementary Material

Li_Tian_et_al._Supplementary_Material.zip
