# Supplementary material for: Antitumor efficacy of liposome-encapsulated NVP-BEZ 235 in combination with irreversible electroporation
Source: Drug Deliv. 2018 Feb 27;25(1):668–78. doi: 10.1080/10717544.2018.1444683 (PMC6058606; doi:10.1080/10717544.2018.1444683)
Supplement: Li_Tian_et_al._Supplementary_Material.zip [file IDRD_A_1444683_SM2144.zip › IRE+LBEZ Hep3B Supplement Data Upload.docx]

**S1 Appendix**

**Verification of L-BEZ disruption and BEZ release from L-BEZ by electroporation**

This set of experiments was designed to evaluate if IRE could disrupt L-BEZ and if it can, what was the lowest field strength that could disrupt L-BEZ.

**Methods**

Electroporation of L-BEZ

Electroporation was carried out using a BTX ECM 830 electroporation system (Harvard Apparatus, Holliston, MA, USA) at various field strengths ranging from 0 to 2500 V/cm. The electroporated L-BEZ was collected, and the particle size was examined via dynamic light scattering at a scatter angle of 90° using a Brookhaven particle size analyzer (Holtsville, NY, USA).

L-BEZ release by electroporation

L-BEZ and a control blank liposome were electroporated using a BTX ECM 830 electroporation system at 2500 V/cm. The supernatant was collected after electroporation. Hep3B human hepatocellular carcinoma cells (obtained from ATCC) were plated in 96-well plates in DMEM. After 24 h, the old cell culture medium was removed from each well, and a mixture of 10 μL supernatant and 100 μL fresh Dulbecco’s modified Eagle’s medium was added to each well. After 72 h of treatment, cell viability was evaluated using a 3-(4,5-dimethylthiazolyl-2)-2,5-diphenyltetrazolium bromide (MTT) assay.

**Results**

Electroporation of L-BEZ

The results of electroporation of L-BEZ are shown in S1 Fig and S1 Table. The particle size was 103 nm before the electroporation, with a narrow polydispersity index (PDI). Phase separation was observed in all electroporated L-BEZ samples. Both the effective diameter and the peak size of the larger particle population increased as the electroporation field strength increased. The PDI also increased as the electroporation field strength increased. The particle size and PDI were the largest at the highest electroporation field strength. These results indicated that destruction of liposomes by electroporation was more effective with increasing field strength.

L-BEZ release by electroporation

We examined whether BEZ was released from L-BEZ by electroporation. The supernatant of electroporated L-BEZ had a cell viability rate of 48.97%, significantly lower than the cell viability rate obtained from the supernatant of the blank liposome (80.12%) (*p* < 0.05, 1-way analysis of variance).

**Discussion**

These results suggest that electroporation disrupted the liposomal particles and that the degree of destruction increased as field strength increased. However, more than 1 distribution of the particle size was observed, even at the lowest electroporation field strength. This observation suggests that liposomes would be disrupted at much lower field strengths than those used in IRE. It also supports our hypothesis that L-BEZ can be disrupted even at the marginal space of the tumor, where it receives less intense electroporation.

However, we were unable to directly monitor the release of BEZ using any spectrometer because BEZ has very weak ultraviolet and fluorescence signals. Thus, the biological effect of BEZ, i.e., its cytotoxicity, was used to evaluate BEZ release. Decreased cell viability was observed in Hep3B cells treated with electroporated L-BEZ supernatant. This could only have been caused by free BEZ in the supernatant, proving that BEZ was indeed released from the liposome by electroporation.

S1 Table. Effective diameter, peak size of the larger particle population, and polydispersity index (PDI) of L-BEZ after electroporation.

| Field Strength  (V/cm) | Effective Diameter  (nm) | Peak Size of the Larger Population  (nm) | PDI |
| --- | --- | --- | --- |
| 0 | 102.9 | --- | 0.085 |
| 250 | 113.9 | 160.0 | 0.121 |
| 500 | 121.3 | 184.7 | 0.152 |
| 1000 | 148.3 | 225.6 | 0.235 |
| 1500 | 167.5 | 306.0 | 0.293 |
| 2000 | 207.4 | 567.1 | 0.305 |
| 2500 | 290.0 | 624.2 | 0.331 |

**S Figures. Effect of electroporation on L-BEZ**. S1 Fig. Electroporation disrupted nanoparticle integrity even at low field strengths. Particle size distributions at electroporation field strengths from 0 to 2500 V/cm. S2 Fig. Drug release from the liposome after electroporation. L-BEZ and blank liposomes were electroporated and the extracts were tested for cytotoxicity against Hep3B cells. L-BEZ had a significantly stronger cytotoxic effect than did blank liposome control (1-way ANOVA followed by Holm-Šidák post hoc, *p* < 0.05).
