# Supplementary material for: Antitumor efficacy of liposome-encapsulated NVP-BEZ 235 in combination with irreversible electroporation
Source: Drug Deliv. 2018 Feb 27;25(1):668–78. doi: 10.1080/10717544.2018.1444683 (PMC6058606; doi:10.1080/10717544.2018.1444683)
Supplement: Li_Tian_et_al._Supplementary_Material.zip [file IDRD_A_1444683_SM2144.zip › S1 Table.docx]

S1 Table. Effective diameter, peak size of the larger particle population, and polydispersity index (PDI) of L-BEZ after electroporation.

| Field Strength  (V/cm) | Effective Diameter  (nm) | Peak Size of the Larger Population  (nm) | PDI |
| --- | --- | --- | --- |
| 0 | 102.9 | --- | 0.085 |
| 250 | 113.9 | 160.0 | 0.121 |
| 500 | 121.3 | 184.7 | 0.152 |
| 1000 | 148.3 | 225.6 | 0.235 |
| 1500 | 167.5 | 306.0 | 0.293 |
| 2000 | 207.4 | 567.1 | 0.305 |
| 2500 | 290.0 | 624.2 | 0.331 |
