# Supplementary figures and images for: Antitumor efficacy of liposome-encapsulated NVP-BEZ 235 in combination with irreversible electroporation
Source: Drug Deliv. 2018 Feb 27;25(1):668–78. doi: 10.1080/10717544.2018.1444683 (PMC6058606; doi:10.1080/10717544.2018.1444683)

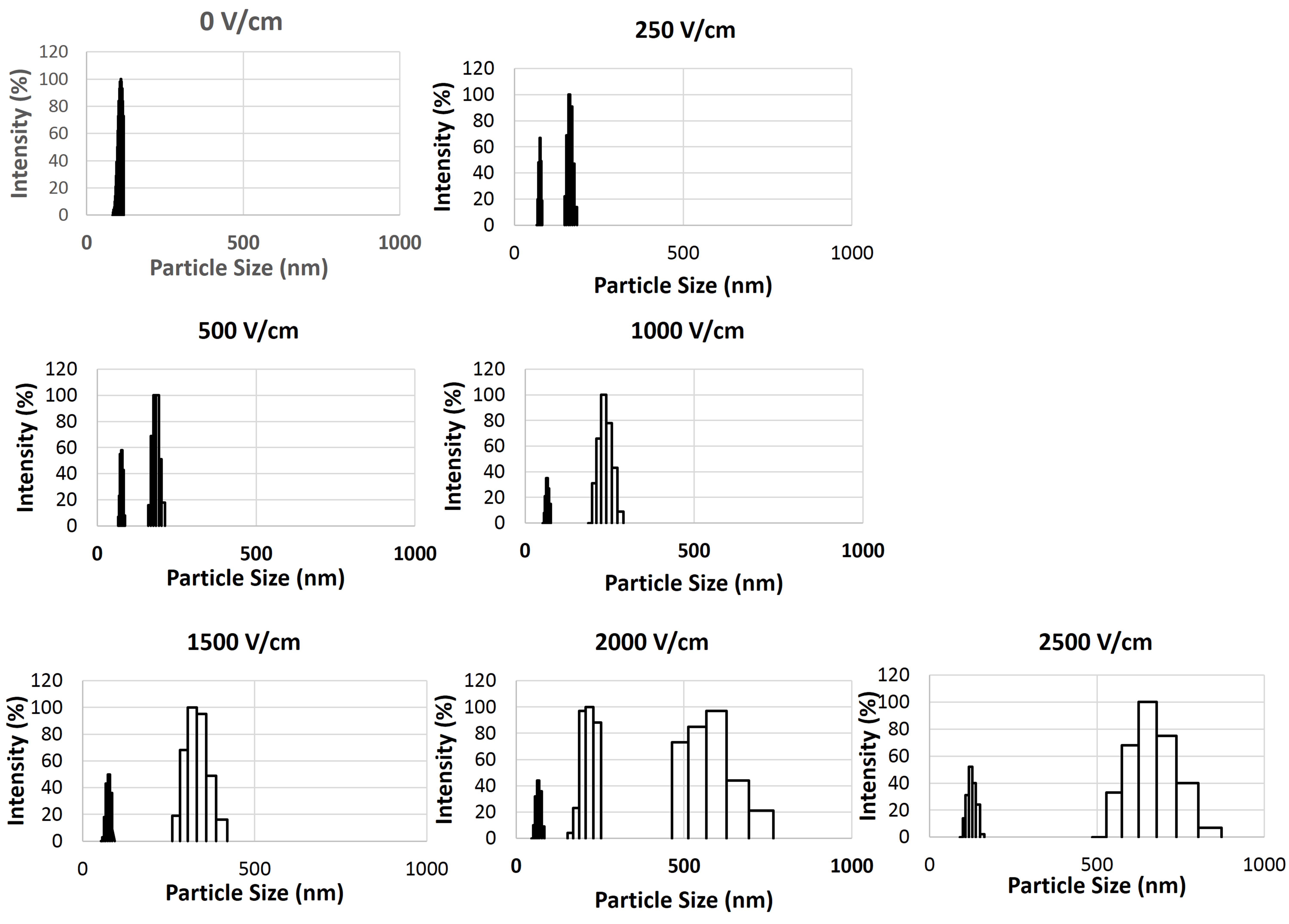

Supplement: Li_Tian_et_al._Supplementary_Material.zip [file IDRD_A_1444683_SM2144.zip › S1 Fig.jpg]

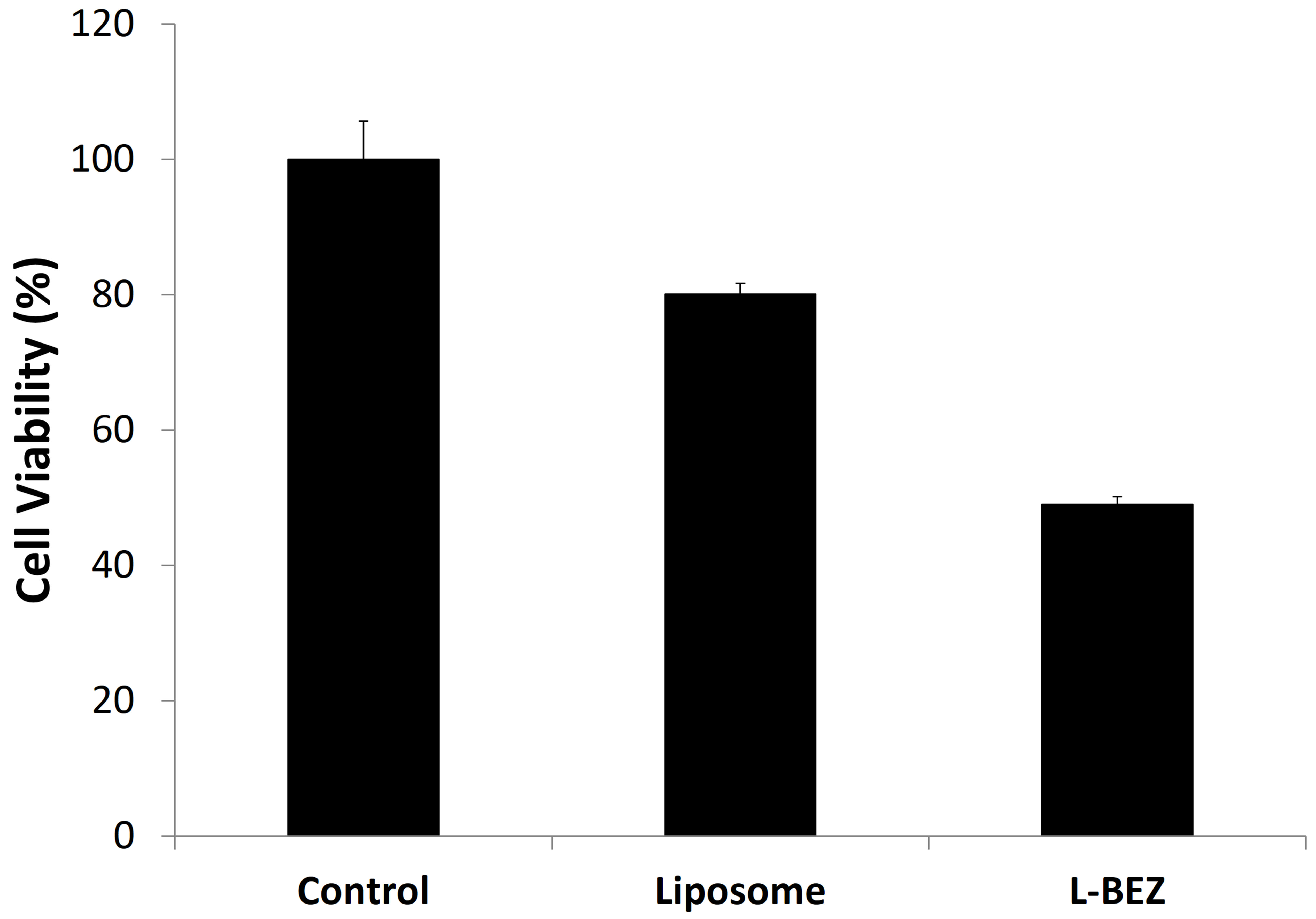

Supplement: Li_Tian_et_al._Supplementary_Material.zip [file IDRD_A_1444683_SM2144.zip › S2 Fig.jpg]
